# Impact of Posterior Vitreous Detachment on Long-Term Functional and Morphological Retinal Status in Patients After Surgical Epiretinal Membrane Removal

**DOI:** 10.3390/jcm15103940

**Published:** 2026-05-20

**Authors:** Alicja Ziontkowska-Wrzałek, Michał Dobrzycki, Anna Machalińska

**Affiliations:** First Department of Ophthalmology, Pomeranian Medical University, Al. Powstańców Wielkopolskich 72, 70-111 Szczecin, Poland; aziontkowskawrzalek@gmail.com (A.Z.-W.); michal.dobrzycki27@gmail.com (M.D.)

**Keywords:** epiretinal membrane, ERM, pars plana vitrectomy, PPV, posterior vitreous detachment, PVD, vitreous status

## Abstract

**Background/Objectives:** Posterior vitreous detachment (PVD), which is closely related to epiretinal membrane (ERM) formation, can affect macular microstructure and function through persistent tractional forces. The aim of this study was to evaluate whether PVD status influences preoperative characteristics and long-term functional and morphological retinal outcomes after ERM surgery. **Methods:** Ninety patients who underwent idiopathic ERM removal were included and divided into two groups on the basis of intraoperative vitreous status: incomplete or complete PVD. Visual function, retinal imaging, microperimetry, and multifocal electroretinography (mfERG) data were assessed preoperatively and at 1, 4, and 12 months postoperatively. **Results:** At baseline, compared with the incomplete PVD group, the complete PVD group demonstrated greater fixation stability and lower variability, along with smaller foveal avascular zone (FAZ) areas in both superficial and deep vascular complexes. In terms of absolute postoperative values, the complete PVD group exhibited superior functional outcomes, including higher macular sensitivity and improved fixation variability parameters at 12 months. Morphologically, the incomplete PVD group showed consistently larger FAZ areas in both superficial and deep vascular complexes. In terms of changes from baseline, best-corrected visual acuity (BCVA) gain was greater in the complete PVD group at 1 and 4 months, with no difference at 12 months, whereas no significant between-group differences were observed for other functional or morphological parameters at any time point. **Conclusions:** Complete PVD is associated with faster visual recovery. Incomplete PVD may induce alterations at the retinal microcirculation level that persist postoperatively and influence microperimetric scores.

## 1. Introduction

Epiretinal membrane (ERM) is a common disorder of the vitreoretinal junction [1] characterized by fibrocellular proliferation on the inner retinal surface [1,2], which may lead to decreased best-corrected visual acuity (BCVA), metamorphopsia, and visual distortion, all of which affect daily activities [3,4]. ERM predominantly affects older individuals [4] and is considered idiopathic (iERM) when it develops in the absence of any associated ocular pathology [1,2,5].

Posterior vitreous detachment (PVD) is an age-related process in which progressive vitreous gel liquefaction and weakening of vitreoretinal adhesion result in separation of the posterior vitreous cortex from the retinal surface. For a normal, uncomplicated PVD to occur, liquefaction of the vitreous gel and vitreoretinal dehiscence must develop in a coordinated manner. Anomalous PVD, as described by Sebag, occurs when vitreous gel liquefaction develops without adequate vitreoretinal dehiscence, leading to persistent vitreoretinal adhesion [6,7]. In some cases, anomalous vitreous separation may result in vitreoschisis, in which splitting of the posterior vitreous cortex leaves its outermost layer adherent to the macula while the remaining vitreous collapses anteriorly [1,6,7]. Increasing evidence suggests that such residual cortical vitreous remnants may be relatively thick, hypercellular, and contractile, generating centripetal tangential traction and contributing to ERM formation [6].

Although the exact pathogenesis of iERM remains incompletely understood [1,4], several mechanisms have been proposed to explain how vitreoretinal interface abnormalities contribute to iERM formation [3,8]. Transient vitreoretinal traction during vitreous separation may induce microdefects in the internal limiting membrane (ILM), facilitating the migration and proliferation of glial and retinal pigment epithelial cells on the retinal surface [1,4]. Additionally, vitreous cortical remnants and hyalocytes remaining adherent to the retina after PVD may undergo proliferation and transdifferentiation, contributing to membrane formation. Furthermore, partial or complete PVD has been observed in approximately 80–95% of eyes with iERM [1], supporting the concept that vitreous separation dynamics are closely associated with disease development.

Pars plana vitrectomy (PPV) with ERM peeling, often combined with ILM removal, is the standard surgical treatment for symptomatic iERM and generally leads to marked anatomical and functional improvement [4]. In selected cases, PVD induction during surgery may be necessary prior to membrane peeling to achieve the intended surgical outcome [9]. Previous studies have shown that surgical induction of PVD may increase tractional stress at the vitreoretinal interface and is associated with a higher risk of intraoperative retinal breaks compared with eyes with pre-existing PVD [10]. An incomplete PVD may render the procedure more technically demanding because of the presence of complex peripheral tractional forces [11]. These forces can also influence macular microstructure and function even before surgery [11]. Therefore, the distinction between complete and incomplete PVD has crucial implications for both surgical strategy and postoperative recovery [9,11].

Our study aimed to investigate the impact of complete versus incomplete PVD, as assessed intraoperatively, on functional and morphological retinal parameters in patients who underwent idiopathic ERM removal with 25-gauge vitrectomy. By combining pre- and postoperative structural imaging with functional assessment, we aimed to determine whether baseline vitreous status influences the final visual and morphological postsurgical outcomes during long-term observation.

To the best of our knowledge, prospective data addressing the prognostic role of vitreous status in iERM surgery remain scarce. The present study expands upon previous research by incorporating a larger patient cohort, extended follow-up, and a comprehensive functional evaluation, including electrophysiological testing in addition to microperimetry, alongside detailed structural assessment. This integrated approach allows for a more thorough characterization of retinal recovery in relation to vitreous status.

## 2. Materials and Methods

This prospective study included 90 patients who underwent surgical removal of the iERM with ILM peeling. All patients completed the 4-month postoperative follow-up, and 58 of the 90 patients completed the 12-month follow-up. The inclusion criteria included the presence of a symptomatic ERM associated with metamorphopsia or resulting in a marked reduction in BCVA, as well as pseudophakia at the time of patient enrolment. Consequently, eyes undergoing combined cataract surgery and PPV were not included in the study. The exclusion criteria included the presence of ocular or retinal pathologies other than ERMs that could affect the validity of the study outcomes.

All patients underwent a conventional 25-gauge three-port PPV performed under peribulbar anaesthesia with 2% lignocaine and 0.5% bupivacaine. PVD status was evaluated intraoperatively on the basis of the configuration of vitreoretinal adhesions in the macular and peripapillary regions, with triamcinolone acetonide used to improve visualization. Complete PVD was defined as complete separation of the posterior hyaloid from the macular and peripapillary regions of the posterior pole, whereas incomplete PVD was defined as persistent vitreoretinal adhesion involving the macular and/or peripapillary regions. In eyes without preexisting PVD, it was induced by engaging the posterior cortical vitreous near the optic disc and gently elevating it in a posteroanterior and peripheral direction. The ERM was completely removed using retinal forceps. Then, the internal limiting membrane (ILM) was stained in a standardized manner using a commercially available dye solution containing 0.18% trypan blue and 0.03% blue dye (TWIN, AL.CHI.MI.A. S.R.L., Ponte San Nicolò, Italy) and peeled using a pinch-and-peel technique. Peripheral vitrectomy was subsequently performed. At the end of the procedure, fluid–air exchange was performed, followed by air–gas exchange using sulphur hexafluoride (SF6). SF6 tamponade was used routinely in all cases as part of a standardized surgical protocol. All surgeries were performed by a single experienced vitreoretinal surgeon.

**Figure 1:**
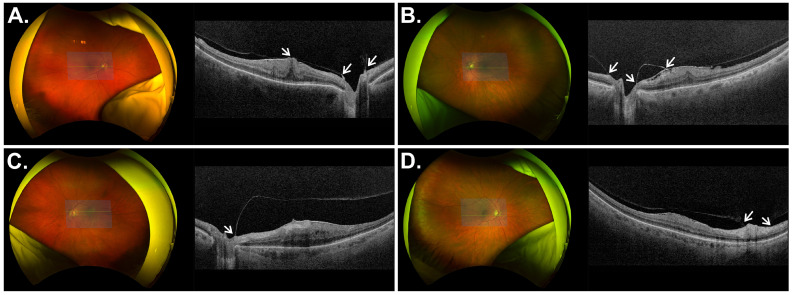
Ultra-widefield fundus photographs and corresponding optical coherence tomography (OCT) B-scans illustrating incomplete posterior vitreous detachment (PVD). (**A**,**B**): Vitreous attachment at both the optic disc and the macula. (**C**,**D**): Incomplete PVD with residual attachment limited to the optic disc and complete release over the macular region. Arrows indicate sites of persistent vitreoretinal adhesion.

On the basis of intraoperative findings regarding vitreous status, the study population was stratified into two groups: an incomplete PVD group and a complete PVD group (Figure 1).

Ophthalmic examinations were conducted in accordance with a standardized protocol implemented in our department and reported previously [12]. In brief, the protocol included enhanced depth imaging optical coherence tomography (EDI-OCT) and optical coherence tomography angiography (OCTA), along with the analyses of retinal thickness in the nine Early Treatment Diabetic Retinopathy Study (ETDRS) subfields (μm), total retinal volume (mm^3^), subfoveal choroidal thickness (µm), choroidal area (mm^2^), and foveal avascular zone (FAZ) area in both the superficial vascular complex (SVC) and deep vascular complex (DVC), expressed in mm^2^.

Similarly, multifocal electroretinography (mfERG) and microperimetry were performed according to the same protocol, with mfERG recordings obtained in accordance with International Society for Clinical Electrophysiology of Vision (ISCEV) standards [13]. Response density (nV/degree^2^) and P1-wave implicit time (ms) were analysed in six concentric rings. Microperimetry was used to assess macular sensitivity as well as fixation stability and variability.

BCVA was assessed using an ETDRS-style chart comprising 40 letters arranged in 8 lines (5 letters per line, 0.1 logMAR progression), with visual acuity ranging from 0.2 to 1.0. Visual acuity was recorded as the number of correctly identified letters, with the results expressed as letter scores and converted to ETDRS-equivalent values by adding 45 letters. Letter-by-letter scoring (0.02 logMAR per letter) was applied, based on the ETDRS method described by [14].

All participants were classified preoperatively according to the Govetto staging system based on optical coherence tomography (OCT) findings [15]. Follow-up examinations were conducted preoperatively and at 1, 4, and 12 months after surgery.

The normality of the data distribution was evaluated using the Shapiro–Wilk test. Owing to the nonnormal distribution of most continuous variables, nonparametric statistical methods were applied. The Mann–Whitney U test was used for between-group comparisons of continuous or ordinal variables, whereas correlations were assessed using Spearman’s rank correlation coefficient (Rs). Categorical variables were analysed using a two-tailed Fisher’s exact test. A *p* value < 0.05 was considered to indicate statistical significance. All analyses were performed using Statistica 13 software.

## 3. Results

### 3.1. Baseline Characteristics of the Study Groups

A total of 90 patients (90 eyes) were included in the study. All patients completed the 4-month postoperative follow-up, and 58 of the 90 patients (64.4%) completed the 12-month follow-up. Loss to follow-up was mainly related to logistical factors and missed scheduled visits. No significant baseline differences were observed between patients who completed the 12-month follow-up and those lost to follow-up (Supplementary Table S1).

On the basis of the intraoperative assessment of vitreous status, the patients were divided into the incomplete PVD group (31 eyes; 34.45%) and the complete PVD group (59 eyes; 65.55%). To explore whether OCT-based preoperative vitreoretinal interface assessment coincided with intraoperative findings we stratified the agreement between preoperative OCT and intraoperative PVD classification. Overall agreement between preoperative OCT and intraoperative findings was 86.7%. Preoperative OCT showed a sensitivity of 86.4% and specificity of 87.1% for identifying complete PVD. The baseline demographic and visual function characteristics of the study groups are summarized in Table 1. No statistically significant differences were observed between the groups in terms of age, sex, or initial BCVA. When initial microperimetry parameters were analysed, greater fixation stability (P1) was observed in the complete PVD group than in the incomplete PVD group (*p* = 0.049). Similarly, fixation variability, expressed as the 63% and 95% bivariate contour ellipse area (BCEA) and the corresponding horizontal semiaxis, was greater in the complete PVD group (detailed values are presented in Table 1). No differences in the values of macular sensitivity (average threshold) were observed between the groups (*p* = 0.91).

Accordingly, no statistically significant differences were observed between the groups in mfERG P1 wave amplitude in R1 (Table 1). Similarly, no differences were detected in the remaining rings (R2–R6). P1 wave implicit time in R1 was significantly longer in the complete PVD group than in the incomplete PVD group (*p* = 0.01).

We subsequently performed a comparative analysis of baseline spectral-domain optical coherence tomography (SD-OCT) ERM characteristics among the groups (Table 2). The groups exhibited no differences in ERM stage according to the Govetto classification, central ETDRS retinal thickness, total retinal volume, subfoveal choroidal thickness, or choroidal area. Similarly, no differences were detected in retinal thickness in the remaining ETDRS sectors (superior, inferior, nasal, or temporal). Interestingly, on OCTA, the FAZ area in the SVC and DVC was significantly larger in the incomplete PVD group than in the complete PVD group (*p* = 0.012 and *p* = 0.013, respectively).

### 3.2. Differences in Postoperative Outcomes and Recovery Dynamics Between the Groups

Postoperative functional and morphological outcomes in the incomplete and complete PVD groups were analysed at the 1-, 4-, and 12-month follow-up visits (Table 3). As the groups differed in selected preoperative functional and morphological measures, postoperative changes in the investigated parameters relative to baseline values were also analysed at each follow-up time point to better distinguish differences related to postoperative recovery dynamics from persistence of pre-existing intergroup disparities.

BCVA improved over time in both groups. In terms of absolute postoperative values, BCVA was greater in the complete PVD group than in the incomplete PVD group at 1 postoperative month (*p* = 0.005), with no difference at 4 and 12 months. Importantly, a significant between-group difference in visual gain was observed at 1 month (*p* = 0.001) and 4 months (*p* = 0.003), whereas no significant differences were found at 12 months, suggesting faster early postoperative visual recovery rather than superior final visual outcome.

The absolute values of macular sensitivity were significantly greater in the complete PVD group at 1 and 12 months (both *p* = 0.01). However, no between-group differences were noted in the relative change compared with the baseline values (*p* = 0.08 at 1 month, *p* = 0.06 at 4 months, and *p* = 0.15 at 12 months), indicating no clear difference in postoperative improvement dynamics between the groups.

Similarly, fixation stability (P1) was significantly greater in the complete PVD group at 1 and 4 months (*p* = 0.003 and *p* = 0.01, respectively), with no significant difference in postoperative change in this parameter compared with the baseline values (*p* = 0.82 at 1 month, *p* = 0.98 at 4 months, and *p* = 0.81 at 12 months), indicating that the postoperative differences were not accompanied by differential postoperative improvement. A similar pattern of changes was observed in relation to fixation variability parameters (63% and 95% BCEA area and horizontal semiaxis), with no significant difference in postoperative change throughout follow-up.

Multifocal ERG analysis revealed that the P1 wave amplitude in R1 was significantly greater in the complete PVD group than in the incomplete PVD group at the 1-month follow-up (*p* = 0.02). However, no differences between the groups were noted in the relative change in the P1 wave amplitude in R1 compared with baseline recordings (*p* = 0.8 at 1 month, *p* = 0.74 at 4 months, and *p* = 0.38 at 12 months).

The FAZ area in both the SVC and DVC remained significantly larger in the incomplete PVD group at all postoperative time points. Importantly, no significant difference in postoperative change was found between the groups at any follow-up interval, suggesting persistence of preoperative intergroup differences.

Central ETDRS retinal thickness was significantly greater in the complete PVD group than in the incomplete PVD group at the 12-month follow-up (*p* = 0.01). No significant between-group differences were observed in the postoperative changes compared with the baseline values at any time point. Accordingly, no differences were observed between the groups at any follow-up visit in total retinal volume, subfoveal choroidal thickness or choroidal area, as expressed both in terms of absolute values and changes relative to baseline measurements.

### 3.3. Correlation Analysis According to PVD Status

Additionally, we investigated the relationships between morphological and functional retinal parameters according to intraoperative PVD status. The FAZ area in both the SVC and DVC was negatively correlated with BCVA across the evaluated time points (Table 4). This correlation was most prominent in relation to the DVC FAZ area in both the incomplete and complete PVD groups, with larger FAZ areas associated with lower BCVA.

We also analysed the correlations between the DVC FAZ area and other functional parameters. In the complete PVD group, a larger DVC FAZ area was associated with lower macular sensitivity (Rs = −0.36, *p* = 0.015 at baseline; Rs = −0.27, *p* = 0.05 at 1 month; Rs = −0.28, *p* = 0.04 at 4 months; and Rs = −0.05, *p* = 0.8 at 12 months). Similar but less prominent correlations were observed between the SVC FAZ area and macular sensitivity (Rs = −0.23, *p* = 0.11 at baseline; Rs = −0.1, *p* = 0.49 at 1 month; Rs = −0.21, *p* = 0.11 at 4 months; and Rs = −0.38, *p* = 0.03 at 12 months).

With respect to the electrophysiological parameters, a larger DVC FAZ area was associated with a lower mfERG P1 wave amplitude, both in the complete PVD group (Rs = −0.27, *p* = 0.07 at baseline; Rs = −0.30, *p* = 0.03 at 1 month; Rs = −0.07, *p* = 0.6 at 4 months; and Rs = −0.36, *p* = 0.04 at 12 months) and in the incomplete PVD group (Rs = −0.04, *p* = 0.8 at baseline; Rs = −0.46, *p* = 0.009 at 1 month; Rs = −0.55, *p* = 0.003 at 4 months; and Rs = +0.23, *p* = 0.3 at 12 months).

Furthermore, the pre- and postoperative BCVA values correlated with objective microperimetry and electrophysiological recordings. In the complete PVD group, higher BCVA corresponded to higher macular sensitivity across all the time points examined (Table 5).

A similar pattern was observed for the fixation parameters: the higher the BCVA was, the greater the fixation stability. Conversely, a lower BCVA corresponded to greater fixation variability, reflected by a larger BCEA (63%) and greater vertical and horizontal semiaxes. A comparable relationship was observed for the 95% BCEA parameters.

## 4. Discussion

The mechanics of adhesion at the vitreoretinal interface play a fundamental role in the development and progression of multiple sight-threatening disorders. Persistent or focal vitreoretinal adhesions may prevent complete posterior vitreous detachment (PVD), allowing tractional forces to be transmitted to the retinal surface and contributing to pathologies such as epiretinal membrane (ERM) formation [5].

The OCT-based classification proposed by the International Vitreomacular Traction Study (IVTS) Group provides a useful framework for interpreting incomplete PVD. By distinguishing vitreomacular adhesion (VMA) from vitreomacular traction (VMT), this system emphasizes that persistent vitreous attachment to the macula may exist even in the absence of marked structural distortion.

In the IVTS classification, VMA represents a specific stage of partial perifoveal PVD characterized by persistent vitreomacular attachment without associated retinal structural abnormalities, whereas progression of PVD may lead to VMT, in which persistent attachment is accompanied by anatomical distortion of the fovea on OCT [16,17].

In the setting of idiopathic ERM, persistent vitreoretinal attachment may represent a biomechanically active state capable of transmitting tractional forces and modulating macular microstructure, rather than a neutral or incidental finding [16].

Notably, even in the absence of clinically evident VMT on OCT, subtle and chronic anteroposterior forces may persist in eyes with partial PVD. Previous descriptions of anomalous PVD have highlighted configurations in which the posterior hyaloid remains apposed to the macula, enabling low-grade but sustained tractional stress. Such traction may not produce obvious foveal deformation detectable on standard SD-OCT, yet it may be associated with microstructural alterations of the internal limiting membrane, facilitate cellular migration, and promote ERM proliferation [8,16].

Given that incomplete PVD may represent a biomechanically active state even in the absence of overt tractional signs on OCT, accurate characterization of the vitreoretinal interface becomes particularly important. Preoperative imaging modalities, including SD-OCT and clinical biomicroscopy, may have limited sensitivity in detecting residual vitreoretinal adhesions or vitreoschisis compared with direct surgical visualization [18]. Therefore, intraoperative assessment of PVD, as performed in the present study, likely reduced the risk of PVD misclassification. OCT-based vitreomacular interface classifications, including the IVTS system [16], may allow partial preoperative characterization of vitreoretinal adhesion patterns [19,20,21]. In the present study, exploratory comparison of preoperative OCT findings with intraoperative PVD classification demonstrated an overall agreement of 86.7%, with a sensitivity of 86.4% and specificity of 87.1% for identifying complete PVD. These findings are comparable to previous reports evaluating OCT-based PVD assessment against intraoperative findings. Hwang et al. reported a sensitivity of 71% and specificity of 88% for OCT detection of complete PVD, while Albabtain et al. reported a sensitivity of 83.3% and specificity of 65.9% for OCT-based assessment of complete PVD compared with intraoperative triamcinolone-assisted evaluation [18,22]. Other studies have also demonstrated a strong correlation between OCT findings and intraoperative visualization of the vitreoretinal interface [23,24]. Nevertheless, OCT-based assessment may still be inconclusive in selected cases, particularly in the presence of subtle residual vitreoretinal adhesions or vitreoschisis [25,26]. Given the good agreement observed between preoperative OCT and intraoperative findings in the present study, further studies are warranted to evaluate the prognostic utility of OCT-based PVD assessment in predicting postoperative recovery patterns.

Our findings indicate that vitreous detachment status is not merely a surgical variable but is associated with distinct preoperative structural and functional retinal characteristics.

The differences in baseline FAZ area between complete and incomplete PVD groups suggest that vitreous status may be associated with differences in the mechanical forces acting on the macula. In eyes with complete PVD, membrane-induced centripetal traction may contribute to displacement and crowding of perifoveal capillaries and consequently to a smaller FAZ in both the superficial and deep vascular plexuses compared to the incomplete PVD group. Such FAZ reduction has been described in idiopathic ERM and is thought to reflect membrane-related tangential traction and macular distortion [27,28,29].

In contrast, persistent vitreoretinal adhesion in eyes with incomplete PVD may be associated with a different pattern of tractional forces, in which combined tangential and anteroposterior forces act simultaneously on the macula, potentially contributing to altered microvascular organization and larger FAZ measurements [6].

Toyama et al. demonstrated that complete PVD was associated with a smaller superficial FAZ area and proposed that persistent vitreous adhesion may generate centrifugal tangential traction contributing to relatively larger FAZ measurements in eyes without complete PVD [30].

Although direct comparisons between complete and incomplete PVD are limited, OCTA studies in idiopathic ERM suggest associations between tractional changes and alterations in vascular patterns within both the superficial and deep capillary plexuses [31,32]. Nevertheless, the precise biomechanical mechanisms underlying these observations remain uncertain.

In addition to microvascular differences, baseline functional assessment revealed more stable fixation in the complete PVD group, as reflected by higher P1 values and lower BCEA. This suggests that the absence of persistent vitreoretinal adhesion may be associated with more stable central visual performance. As shown by Sadda et al., fixation stability parameters assessed by microperimetry may vary according to the presence or resolution of vitreomacular adhesion [33]. Contrary to our results, Serino et al., using an intraoperative distinction between complete and incomplete PVD, demonstrated that eyes with incomplete PVD exhibited worse baseline visual acuity, higher Govetto stage, and greater central retinal thickness compared with eyes with complete PVD, whereas FAZ and microperimetric parameters did not significantly differ between groups [11]. Notably, their study was conducted in a much smaller cohort and had a multicenter design. On the contrary our study group was much larger, and all surgeries were performed solely by one vitreoretinal surgeon. It is worth mentioning that we observed significant negative correlations between functional measures and FAZ areas at baseline and throughout follow-up, indicating that larger FAZ areas were associated with lower BCVA, reduced macular sensitivity, and impaired bioelectrical function. Previous studies have shown that changes in FAZ morphology may be associated with impaired visual function and may serve as potential biomarkers of retinal functional status in macular diseases. Consequently, anatomical disruption, vascular remodeling, and functional impairment in idiopathic ERM appear to be closely interconnected [34]. This finding suggests a close association between functional status and the microvascular architecture of the macula and may partially explain the discrepancies between our results and previously published data. Interestingly, Chung et al. classified eyes according to whether PVD had to be surgically induced during vitreous procedures and found no significant differences in baseline BCVA between the groups [9], which is consistent with our findings.

Postoperative analysis of our data revealed a consistent pattern of intergroup differences across functional and microvascular parameters. Although ETDRS visual acuity improved in both groups, a significant between-group difference in visual gain was observed only at 1 and 4 months, indicating a faster early functional recovery in the complete PVD group. However, by the 12-month follow-up, visual acuity outcomes were comparable between the groups. These findings align with the prospective study by Serino et al. [11], in which the intergroup difference in visual acuity persisted until postoperative month 3. This pattern suggests that vitreous configuration may be associated primarily with differences in the kinetics of early functional recovery rather than final visual outcome. In line with this observation, Chung et al. did not identify significant differences in final postoperative BCVA between groups, although the duration of follow-up in that study was not clearly specified [9].

It is worth noting that previous studies primarily focused on absolute postoperative values and longitudinal parameter trajectories between groups [9,11]. In contrast, our study additionally evaluated postoperative changes relative to baseline measurements, allowing differentiation between persistent baseline disparities and true differences in postoperative recovery dynamics.

Importantly, several postoperative intergroup differences, particularly those related to FAZ measurements and fixation parameters, should be interpreted in the context of the significant baseline differences observed between the groups. Although the FAZ area in both superficial and deep vascular plexuses remained significantly larger in the incomplete PVD group throughout follow-up, no significant between-group differences were observed in postoperative change relative to baseline values, suggesting persistence of pre-existing microvascular disparities rather than divergent postoperative remodeling. Similarly, while macular sensitivity, fixation stability, and BCEA favored the complete PVD group at multiple time points, postoperative changes relative to baseline were generally comparable between the groups. In contrast, conventional structural parameters, including total retinal volume and choroidal metrics, did not differ significantly between groups.

A limitation of the present study is the reduced patient retention at the 12-month follow-up visit, which may have reduced the statistical power of long-term analyses. However, no significant baseline differences were observed between completers and non-completers, suggesting that the impact of attrition on the long-term analyses was likely limited.

In conclusion, incomplete PVD may be associated with alterations at the level of retinal microcirculation, potentially affecting macular function. Our findings indicate that visual recovery may occur more rapidly in eyes with complete PVD. Importantly, the present study provides a long-term perspective on these structure–function relationships. Although surgical removal of the membrane eliminates traction, the preoperative status of the vitreoretinal interface may reflect different degrees of chronic traction-related retinal remodeling, including microvascular and foveal alterations that may contribute to postoperative differences in selected structural and functional parameters despite comparable final visual outcomes.

## Figures and Tables

**Table 1:** Baseline demographic and functional characteristics according to intraoperative PVD status. Statistically significant values are highlighted in bold.

Parameters	Incomplete PVD	Complete PVD	*p*-Value
N (%)	31 (34.45)	59 (65.55)	
Age (years)			0.109
Median (IQR)	74 (8)	73 (8)	
Mean ± SD	74.1 ± 6.27	71.6 ± 5.3	
Sex (F/M)	17/14	36/23	0.65
BCVA			0.249
Median (IQR)	68 (8)	67 (11)	
Mean ± SD	66.52 ± 9.5	64.17 ± 10.06	
Average threshold [dB]			0.912
Median (IQR)	24.4 (3.7)	24.3 (3.1)	
Mean ± SD	23.67 ± 3.01	23.99 ± 2.47	
Fixation Stability P1 [%]			**0.049**
Median (IQR)	96 (7)	98 (9)	
Mean ± SD	92.61 ± 8.99	94.14 ± 9.28	
63% BCEA: area [deg^2^]			**0.047**
Median (IQR)	0.6 (0.9)	0.3 (1)	
Mean ± SD	1.14 ± 1.26	2 ± 7.99	
95% BCEA: area [deg^2^]			**0.043**
Median (IQR)	1.9 (2.6)	1 (3.1)	
Mean ± SD	3.4 ± 3.76	5.99 ± 23.93	
63% BCEA horizontal semiaxis [deg]			**0.037**
Median (IQR)	1 (0.6)	0.8 (0.6)	
Mean ± SD	1.1 ± 0.48	1.08 ± 1.61	
95% BCEA horizontal semiaxis [deg]			**0.037**
Median (IQR)	1.7 (1.1)	1.4 (1)	
Mean ± SD	1.89 ± 0.84	1.88 ± 2.79	
P1 wave amplitude in R1 [nV/degree^2^]			0.111
Median (IQR)	57.47 (40.39)	72.15 (40.9)	
Mean ± SD	62.92 ± 29.14	73.79 ± 27.81	
P1 wave implicit time in R1 [ms]			**0.01**
Median (IQR)	45.1 (5.8)	49.0 (5.9)	
Mean ± SD	44.44 ± 6.51	47.39 ± 4.51	

Fisher’s exact test for categorical variables or the Mann–Whitney U test for continuous and ordinal variables. IQR, interquartile range.

**Table 2:** Baseline morphological characteristics according to intraoperative PVD status. Statistically significant values are highlighted in bold.

Parameters	Incomplete PVD	Complete PVD	*p*-Value
Central ETDRS retinal thickness [μm]			0.075
Median (IQR)	467 (118)	509 (116)	
Mean ± SD	475.32 ± 89.89	506.02 ± 78.66	
Total retinal volume [mm^3^]			0.839
Median (IQR)	10.51 (2.31)	10.98 (1.45)	
Mean ± SD	11.13 ± 1.69	10.92 ± 1.04	
FAZ area in SVC [mm^2^]			**0.012**
Median (IQR)	0.17 (0.1)	0.08 (0.16)	
Mean ± SD	0.21 ± 0.17	0.14 ± 0.14	
FAZ area in DVC [mm^2^]			**0.013**
Median (IQR)	0.29 (0.26)	0.19 (0.17)	
Mean ± SD	0.4 ± 0.32	0.25 ± 0.2	
Subfoveal choroidal thickness [μm]			0.845
Median (IQR)	233 (122)	244 (106)	
Mean ± SD	246.9 ± 90.91	245.03 ± 80.71	
Choroidal area [mm^2^]			0.649
Median (IQR)	1.55 (0.76)	1.57 ± 0.63	
Mean ± SD	1.67 ± 0.51	1.58 ± 0.44	

Fisher’s exact test for categorical variables or the Mann–Whitney U test for continuous and ordinal variables. IQR, interquartile range.

**Table 3:** Postoperative morphological and functional outcomes at the 1-, 4-, and 12-month follow-ups according to intraoperative PVD status. The *p*-value refers to comparisons of postoperative changes from baseline between groups at each follow-up visit. Statistically significant values are highlighted in bold.

Parameters	Incomplete PVD Median (IQR) Mean ± SD			Complete PVD Median (IQR) Mean ± SD			*p*-Value		
	**1 Month**	**4 Months**	**12 Months**	**1 Month**	**4 Months**	**12 Months**	**1 Month**	**4 Months**	**12 Months**
BCVA	70 (12) 68.19 ± 9.16	74 (9) 72.29 ± 8.9	76 (11) 75.74 ± 7.24	74 (11) 71.93 ± 8.44	76 (9) 75.64 ± 6.45	80 (11) 77.74 ± 6.8	**0.001**	**0.003**	0.11
Average threshold [dB]	23.6 (2.6) 23.11 ± 2.8	24.7 (3.8) 23.85 ± 2.97	24.3 (3.3) 23.45 ± 4.53	24.95 (2.15) 24.42 ± 2.28	25.5 (3) 25.01 ± 2.16	25.9 (2.7) 25.9 ± 2.11	0.08	0.06	0.15
Fixation Stability P1 [%]	96 (5) 94.31 ± 6.9	97 (5) 96.21 ± 3.3	97 (4) 96.12 ± 4.05	99 (3) 96.59 ± 7.27	98.5 (3) 97.64 ± 3.51	99 (4) 97.35 ± 4.1	0.82	0.98	0.81
63% BCEA: area [deg^2^]	0.7 (0.5) 0.82 ± 0.78	0.5 (0.4) 0.57 ± 0.36	0.5 (0.5) 0.68 ± 0.49	0.3 (0.3) 0.62 ± 1.29	0.35 (0.3) 0.42 ± 0.43	0.3 (0.3) 0.42 ± 0.45	0.65	0.84	0.43
95% BCEA: area [deg^2^]	2 (1.3) 2.46 ± 2.3	1,4 (1.6) 1.72 ± 1.08	1,5 (1.2) 2.01 ± 1.48	0.8 (0.9) 1.83 ± 3.84	1.05 (1.1) 1.27 ± 1.29	1 (0.9) 1.26 ± 1.35	0.72	0.86	0.46
63% BCEA horizontal semiaxis [deg]	0.9 (0.4) 0.99 ± 0.38	0.9 (0.2) 0.91 ± 0.33	0.9 (0.4) 0.91 ± 0.32	0.6 (0.4) 0.71 ± 0.45	0.7 (0.4) 0.72 ± 0.34	0.6 (0.5) 0.66 ± 0.32	0.38	0.91	0.75
95% BCEA horizontal semiaxis [deg]	1.6 (0.7) 1.73 ± 0.63	1.5 (0.5) 1.58 ± 0.57	1.5 (0.6) 1.57 ± 0.55	1.1 (0.6) 1.24 ± 0.77	1.15 (0.7) 1.26 ± 0.58	1 (0.8) 1.16 ± 0.54	0.28	0.78	0.63
FAZ area in SVC [mm^2^]	0.13 (0.13) 0.17 ± 0.17	0.15 (0.13) 0.22 ± 0.23	0.2 (0.15) 0.23 ± 0.14	0.08 (0.07) 0.11 ± 0.1	0.1 (0.08) 0.12 ± 0.1	0.08 (0.07) 0.11 ± 0.08	0.33	0.39	0.15
FAZ area in DVC [mm^2^]	0.25 (0.16) 0.29 ± 0.21	0.23 (0.25) 0.29 ± 0.26	0.16 (0.21) 1.32 ± 5.47	0.15 (0.11) 0.19 ± 0.18	0.14 (0.09) 0.16 ± 0.09	0.1 (0.05) 0.11 ± 0.05	0.61	0.61	0.33
Central ETDRS retinal thickness [μm]	419 (74) 425.81 ± 65.34	396.5 (70) 403.13 ± 61,95	372 (51) 375.96 ± 47,29	437 (50) 443.58 ± 51.22	414 (52) 422.37 ± 53.35	404 (39) 403.89 ± 44	0.31	0.28	0.81
Total retinal volume [mm^3^]	9.92 (0.98) 9.85 ± 0.7	9.48 (0.94) 9.48 ± 0.7	9.02 (0.67) 9.13 ± 0.65	9.95 (0.85) 9.84 ± 0.74	9.46 (0.92) 9.43 ± 0.68	9.12 (0.99) 8.99 ± 0.76	0.98	0.77	0.8
Subfoveal choroidal thickness [μm]	235 (99) 240.13 ± 79.62	216.5 (83) 228.5 ± 71.54	198 (65) 212.16 ± 67.88	232 (124) 238.47 ± 75.5	222 (109) 232.85 ± 65.4	230 (93) 233.11 ± 64.66	0.89	0.9	0.95
Choroidal area [mm^2^]	1.51 (0.75) 1.58 ± 0.45	1.47 (0.65) 1.55 ± 0.42	1.33 (0.32) 1.47 ± 0.4	1.49 (0.66) 1.55 ± 0.43	1.52 (0.59) 1.53 ± 0.41	1.56 (0.51) 1.53 ± 0.4	0.19	0.64	0.59
P1 wave amplitude in R1 [nV/degree^2^]	61.09 (43.13) 62.92 ± 29.14	74.29 (39.28) 69.97 ± 27.39	76.51 (37.98) 67.33 ± 24.42	74.66 (20.76) 75.23 ± 20.55	72.34 (39.31) 80.03 ± 35.88	75 (46.44) 76.86 ± 28.8	0.8	0.74	0.38
P1 wave implicit time in R1 [ms]	50 (5.9) 48.86 ± 4.78	47.1 (4.9) 47.98 ± 5.11	47.1 (4.9) 48.12 ± 6.12	50 (6.8) 49.27 ± 4.37	49 (4.9) 48.3 ± 4.45	48 (6.9) 46.74 ± 4.64	0.09	0.12	0.06

Mann–Whitney U test. IQR, interquartile range.

**Table 4:** Correlations between the FAZ area and BCVA in the incomplete and complete PVD groups. Statistically significant values are highlighted in bold.

Correlation	Incomplete PVD (Rs)				Complete PVD (Rs)			
	Baseline	1 Month	4 Months	12 Months	Baseline	1 Month	4 Months	12 Months
BCVA vs SVC FAZ area	+0.31	−0.01	**−0.4**	−0.24	+0.09	+0.06	−0.09	−0.13
BCVA vs. DVC FAZ area	**−0.43**	**−0.37**	**−0.46**	−0.08	**−0.31**	**−0.27**	**−0.27**	**−0.4**

Test for Spearman rank correlation coefficient.

**Table 5:** Correlations between selected functional parameters in the incomplete and complete PVD groups. Statistically significant values are highlighted in bold.

Correlation	Incomplete PVD (Rs)				Complete PVD (Rs)			
	Baseline	1 Month	4 Months	12 Months	Baseline	1 Month	4 Months	12 Months
BCVA vs. Average Threshold	+0.06	+0.26	+0.16	**+0.42**	**+0.5**	**+0.46**	**+0.67**	**+0.57**
BCVA vs. Fixation Stability (P1)	+0.09	+0.03	+0.07	−0.02	**+0.41**	**+0.32**	**+0.34**	+0.16
BCVA vs. Fixation Stability (P2)	+0.14	+0.17	+0.03	+0.11	**+0.36**	**+0.34**	+0.19	+0.09
BCVA vs. 63% BCEA (area/vertical/horizontal)	−0.16/−0.25/−0.05	−0.07/−0.11/−0.09	−0.06/−0.01/−0.12	+0.08/+0.09/+0.1	**−0.41/−0.39/−0.4**	**−0.38**/**−0.27**/**−0.39**	**−0.26**/−0.24/**−0.26**	−0.06/−0.21/−0.01
BCVA vs. 95% BCEA (area/vertical/horizontal)	−0.17/−0.26/−0.06	−0.07/−0.11/−0.05	−0.06/+0.02/−0.15	+0.11/+0.07/+0.09	**−0.41/−0.37/−0.4**	**−0.36**/**−0.27**/**−0.4**	**−0.28**/−0.22/**−0.27**	−0.09/−0.17/+0.01
BCVA vs. P1 wave amplitude in R1	**+0.41**	**+0.36**	+0.34	+0.21	**+0.31**	+0.02	**+0.3**	+0.06

Test for the Spearman rank correlation coefficient.

## Data Availability

The data that were used to support the findings of this study are available from the corresponding author upon request.

## Supplementary Materials

The following supporting information can be downloaded at: https://www.mdpi.com/article/10.3390/jcm15103940/s1, Table S1: Comparison of baseline characteristics between completers and non-completers at 12-month follow-up. Statistically significant values are highlighted in bold.
