# Supplementary material for: Impact of Posterior Vitreous Detachment on Long-Term Functional and Morphological Retinal Status in Patients After Surgical Epiretinal Membrane Removal
Source: J Clin Med. 2026 May 20;15(10):3940. doi: 10.3390/jcm15103940 (PMC13207993; doi:10.3390/jcm15103940)
Supplement: Supplementary file 1 [file jcm-15-03940-s001.zip › jcm-4295425-supplementary.pdf]

Supplementary Table S1. Comparison of baseline characteristics between completers and non-completers at 12-month follow-up. Statistically significant values are highlighted in bold.

Parameters	Completers	Non-completers	p-value
N (%)	58 (64.45)	32 (35.55)	
Age (years)<br>Median (IQR)<br>Mean ± SD	 73 (7)<br>72.6 ± 4.86	 74 (8.5)<br>73.19 ± 7.05	0.64
Sex (F / M)	32 / 26	21 / 11	0.38
BCVA<br>Median (IQR)<br>Mean ± SD	 67 (11)<br>65.55 ± 9.52	 67.5 (12)<br>63.94 ± 10.58	0.52
Average threshold [dB]<br>Median (IQR)<br>Mean ± SD	 24.2 (3.1)<br>24.05 ± 2.48	 24.45 (2.95)<br>23.58 ± 2.96	0.7
Fixation Stability P1 [%]<br>Median (IQR)<br>Mean ± SD	 97.5 (7)<br>94.59 ± 7.01	 97 (12)<br>91.84 ± 12.06	0.72
63% BCEA: area [deg^2]<br>Median (IQR)<br>Mean ± SD	 0.45 (0.7)<br>0.83 ± 0.91	 0.5 (1.6)<br>3.28 ± 10.77	0.64
95% BCEA: area [deg^2]<br>Median (IQR)<br>Mean ± SD	 1.4 (2.1)<br>2.49 ± 2.75	 1.6 (4.8)<br>9.83 ± 32.25	0.59
63% BCEA horizontal semiaxis [deg]<br>Median (IQR)<br>Mean ± SD	 0.8 (0.5)<br>0.9 ± 0.44	 0.85 (0.75)<br>1.43 ± 2.13	0.37
95% BCEA horizontal semiaxis [deg]<br>Median (IQR)<br>Mean ± SD	 1.45 (0.9)<br>1.56 ± 0.75	 1.5 (1.3)<br>2.47 ± 3.69	0.36
P1 wave amplitude in R1 [nV/degree^2]<br>Median (IQR)<br>Mean ± SD	 65.77 (44.46)<br>68.11 ± 30.53	 72.05 (33.93)<br>74.76 ± 22.19	0.23
P1 wave implicit time in R1 [ms]<br>Median (IQR)<br>Mean ± SD	 47.1 (6.9)<br>45.77 ± 6.1	 47.1 (5.4)<br>47.46 ± 3.82	0.4
Central ETDRS retinal thickness [μm]<br>Median (IQR)<br>Mean ± SD	 494.5 (99)<br>494.17 ± 85.47	 508.5 (139)<br>497.75 ± 81.07	0.52
Total retinal volume [mm^3]<br>Median (IQR)<br>Mean ± SD	 10.98 (1.45)<br>10.94 ± 1.17	 10.94 (2.02)<br>11.08 ± 1.5	0.89
FAZ area in SVC [mm^2]<br>Median (IQR)<br>Mean ± SD	 0.12 (0.13)<br>0.16 ± 0.14	 0.14 (0.19)<br>0.19 ± 0.19	0.72
FAZ area in DVC [mm^2]<br>Median (IQR)<br>Mean ± SD	 0.24 (0.25)<br>0.29 ± 0.24	 0.21 (0.2)<br>0.34 ± 0.3	0.42
Subfoveal choroidal thickness [μm]<br>Median (IQR)<br>Mean ± SD	 233.5 (114)<br>247.03 ± 78.23	 246 (112.5)<br>243.22 ± 94.48	0.84

Choroidal area [mm^2]<br>Median (IQR)<br>Mean ± SD	 1.57(0.57)<br>1.62 ± 0.43	 1.56 ± 0.79<br>1.6 ± 0.54	 0.88

Fisher's exact test for categorical variables or the Mann–Whitney U test for continuous and ordinal variables. IQR, interquartile range.
